# Uncovering Distinct Peptide Charging Behaviors in Electrospray Ionization Mass Spectrometry Using a Large-Scale Dataset

**DOI:** 10.1021/jasms.3c00325

**Published:** 2023-12-14

**Authors:** Allyn M. Xu, Lauren C. Tang, Marko Jovanovic, Oded Regev

**Affiliations:** † Department of Mathematics, Courant Institute of Mathematical Sciences, New York University, New York, New York 10012, United States; ‡ Department of Biological Sciences, Columbia University, New York, New York 10027, United States; § Computer Science Department, Courant Institute of Mathematical Sciences, New York University, New York, New York 10012, United States

## Abstract

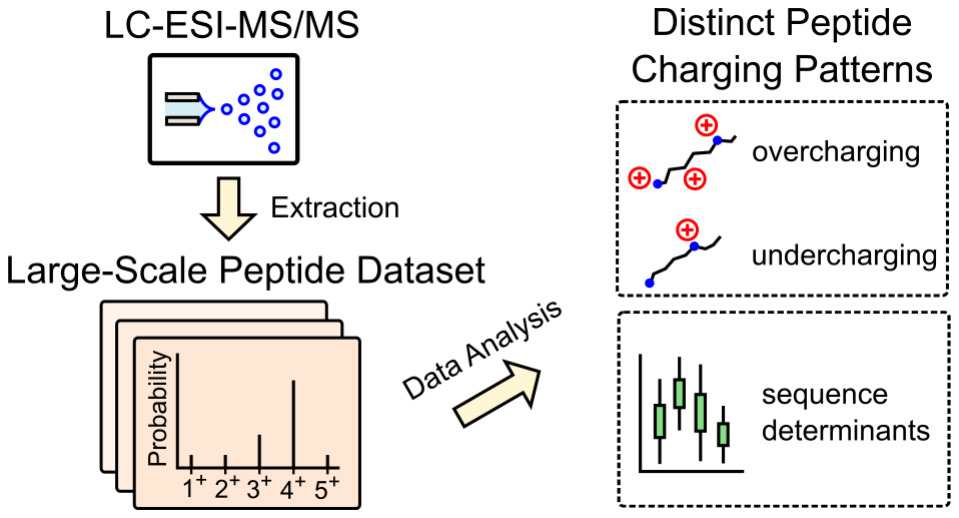

Electrospray ionization is a powerful and prevalent technique used to ionize analytes in mass spectrometry. The distribution of charges that an analyte receives (charge state distribution, CSD) is an important consideration for interpreting mass spectra. However, due to an incomplete understanding of the ionization mechanism, the analyte properties that influence CSDs are not fully understood. Here, we employ a machine learning-based approach and analyze CSDs of hundreds of thousands of peptides. Interestingly, half of the peptides exhibit charges that differ from what one would naively expect (the number of basic sites). We find that these peptides can be classified into two regimes (undercharging and overcharging) and that these two regimes display markedly different charging characteristics. Notably, peptides in the overcharging regime show minimal dependence on basic site count, and more generally, the two regimes exhibit distinct sequence determinants. These findings highlight the rich ionization behavior of peptides and the potential of CSDs for enhancing peptide identification.

## Introduction

Over the years, electrospray ionization^1^ (ESI) has become a leading ionization technique for pairing with liquid chromatography tandem mass spectrometry (LC–MS/MS).^2,3^ ESI’s ability to ionize a wide range of biomolecules and to process samples in a high-throughput manner^4−8^ has greatly broadened the scope of mass spectrometry,^9,10^ enabling applications to proteomics,^11−14^ clinical biology,^15,16^ drug discovery,^17^ and more.^18^

ESI ionizes aqueous solutions through maintaining a high voltage potential across a capillary, which vaporizes the solution into a mist of highly charged droplets.^19^ As the solvent continues to evaporate, the droplets experience an increased charge density and fissure into smaller droplets upon reaching their Rayleigh limit,^20^ the point at which the Coulombic forces overcome the surface tension. The charges are ultimately deposited onto the analytes, through mechanisms that are still not fully understood,^3,21^ producing gaseous ionic molecules that arrive at the mass analyzer. Several theories have been proposed to explain the ionization mechanism, such as the ion evaporation model,^22,23^ the charge residue model,^24,25^ and the chain-ejection model,^26,27^ which differ in their assumptions of how the analyte interacts with the droplet. It is believed that these evaporation models are appropriate for different types of analytes, depending on their size and structure.^27^ However, the dynamic nature of ESI and the inability to directly observe ESI at the molecular scale have made it challenging to fully characterize the determinants of analyte ionization.

Here we utilize a data-driven approach to investigate the ESI ionization of peptides. We reasoned that a systematic analysis of a large-scale dataset would not only complement existing studies on select analytes but also provide more insights than previous “black box”^28^ deep learning approaches.^29^ We therefore generated a dataset containing charging information on hundreds of thousands of peptides, using both new and published LC–MS/MS runs. For each peptide, the resulting dataset includes the measured charge state distribution (CSD), defined as the relative intensities of the ions produced by that peptide.

We next employed machine learning on this dataset to gain insights into the relationship between peptide sequence and CSD. Our analysis revealed that half of the peptides exhibited charges that differed from the number of basic sites (arginine, lysine, histidine, and unmodified N-terminus). Classifying these peptides into two regimes, namely, undercharging and overcharging, we identified striking differences in their ionization characteristics. Specifically, we observed that for overcharged peptides, mass takes precedence over basic site count and that charging in the two regimes is affected by distinct amino acid features.

Overall, our findings offer new insights into the complex dynamics of peptide ionization, highlight the fact that CSDs contain rich information about peptide sequences, and may open opportunities for applications to identification pipelines in proteomics.

## Methods

### Sample Preparation and Mass Spectrometry

To include LC–MS/MS runs with different experimental parameters, we ran our own experiments varying the ESI voltage, gradient length, and sample flow rate.

HeLa cells were grown to 90% confluency and washed twice with PBS before direct lysis on the plate. Total proteins were extracted using a urea lysis buffer (8 M urea, 75 mM NaCl, 50 mM Tris/HCl, pH 8.0, 1 mM EDTA). The protein concentration was determined by Pierce BCA assay. 20 μg of total protein was processed further. Disulfide bonds were reduced with 5 mM dithiothreitol and cysteines were subsequently alkylated with 10 mM iodoacetamide. Samples were diluted 1:4 with 50 mM Tris/HCl (pH 8.0), and sequencing grade modified trypsin (Promega) was added in an enzyme-to-substrate ratio of 1:50. After 16 h of digestion, the samples were acidified with 1% formic acid (final concentration). Tryptic peptides were desalted on C18 StageTips according to Rappsilber et al.^30^ and evaporated to dryness in a vacuum concentrator. Desalted peptides were reconstituted in buffer A (3% acetonitrile and 0.2% formic acid).

For mass spectrometer runs that were run in April 2021, 2 μg of peptides was analyzed on a Thermo Scientific Orbitrap Q Exactive HF mass spectrometer coupled via a 25 cm long, 1.6 μm particle size Aurora C18 column (IonOpticks) to an Acuity M Class UPLC system (Waters). For the long gradient, peptides were separated at a flow rate of 400 nL/min with a linear gradient spanning 2 min from 5% to 8% solvent B (100% acetonitrile and 0.1% formic acid), followed by an 87 min linear gradient from 8% to 22% solvent B, a 20 min linear gradient from 22% to 30% solvent B, a 14 min linear gradient from 30% to 60% solvent B, and a 1 min linear gradient from 60% to 90% solvent B. Each sample was run for 160 min, including sample loading and column equilibration times. For the short gradient, peptides were separated at a flow rate of 400 nL/min in linear steps from 2% to 8% solvent B over 1 min, from 8% to 30% solvent B over 33 min, from 30% to 60% solvent B over 5 min, and from 60% to 90% solvent B over 5 min. Each sample was run for 90 min, including sample loading and column equilibration times.

For mass spectrometer runs that were run in August 2021, 2 μg of peptides was analyzed on a Thermo Scientific Orbitrap Q Exactive HF mass spectrometer coupled via a 15 cm long, 3 μm particle size EASY-Spray C18 column (ThermoFisher Scientific) to an Acuity M Class UPLC system (Waters). Peptides were separated at varying flow rates ranging from 200 nL/min to 800 nL/min in 200 nL/min increments. The peptides were separated in linear steps from 5% to 8% solvent B over 4 min, from 8% to 14% solvent B over 45 min, from 14% to 22% solvent B over 45 min, from 22% to 30% solvent B over 20 min, from 30% to 60% solvent B over 9 min, and from 60% to 90% solvent B over 1 min. Each sample was run for 190 min including sample loading and column equilibration times.

Data were acquired in data dependent mode using the Xcalibur 4.1 software. MS1 spectra were measured with a resolution of 120 000, an AGC target of 3e6, and a mass range from 300 to 1800 *m*/*z*. Up to 12 MS2 spectra per duty cycle were triggered at a resolution of 15 000, an AGC target of 1e5, an isolation window of 1.6 *m*/*z*, and a normalized collision energy of 28.

### Gathering Published LC–MS/MS Raw Files

To supplement our LC–MS/MS runs, we gathered published raw files from two sources: Confetti^31^ and Meier et al.^32^ These raw files are located at the ProteomeXchange Consortium via PRIDE partner repository^33^ with data identifier PXD000900 (Confetti) and PXD019086 (Meier et al.).

### Database Search Parameters and Acceptance Criteria for Identifications

To identify peptides that were present in the LC–MS/MS runs, the raw LC–MS/MS files were analyzed with the MaxQuant software^34^ version 1.6.10.43 (ours, Confetti) or obtained from the original publication (Meier et al.). MaxQuant was run with default parameters: maximum of two missed cleavages, methionine oxidation, and N-terminal acetylation as variable modifications, minimum peptide length of 7 amino acids, maximum peptide mass of 4600 Da, first search mass tolerance of 20 ppm, MS/MS match tolerance of 20 ppm for fragment ions, and a false discovery rate of 1% at both the peptide spectrum match and protein level. As a fixed modification, cysteine carbamidomethylation (ours) or *N*-ethylmaleimidation (Confetti) were used. The enzymes used were trypsin (ours) and ArgC, AspN, chymotrypsin, GluC, LysC, trypsin (Confetti). The search was performed against the *Homo sapiens* Uniprot database (accessed May 2017; 71 567 entries).

### Preprocessing Steps for LC–MS/MS Raw Files

Preprocessing steps were performed to access the MS1 profile peaks from the raw LC–MS/MS files. The Thermo.raw files from Orbitrap runs were converted to .mzML format using MSConvert from ProteoWizard version 3.0,^35^ with the vendor peak picking setting enabled to obtain centroided MS1 peaks. The MS1 spectra of Bruker .d folders from timsTOF runs were accessed using alphatims v. 1.0.0.^36^ The MS1 profile peaks were centroided using our own procedure (Supplementary Text 2). These centroided MS1 peaks were fed into our downstream CSD extraction scheme.

### CSD Extraction Scheme

Per-scan CSD readings were extracted from MS1 scans for MS2-identified peptides using the following scheme. For each peptide and each charge state from 1^+^ to 5^+^, relevant MS1 scans were searched for peaks that matched the theoretical isotope envelope and passed stringent filtering requirements. From these peaks, the charge state intensities were estimated and then normalized to obtain the peptide’s CSD reading for that scan. Finally, these per-scan CSDs were combined into one CSD estimate per peptide by performing an intensity-weighted average across scans. The full details of the extraction scheme are provided in Supplementary Text 2.

One of the main design goals was to achieve high quality CSD readings. As such, we applied stringent filtering and retained only CSD readings that contained confident intensity estimates for all five charge states. To determine whether a peptide’s charge state was confidently present in an MS1 spectrum, we verified that (i) the theoretical isotope peaks had low *m*/*z* offset from the observed isotope peaks, (ii) the shape of the theoretical isotope distribution matched the observed isotope distribution (cosine similarity >0.98), and (iii) there was an absence of peaks that might belong to the isotope distributions of other peptides (chimeric peaks). On the other hand, a charge state was denoted absent and thereby assigned an extracted intensity of 0 if no peaks were in a sufficiently large *m*/*z* vicinity. Through tuning of the thresholds used for filtering, the extraction scheme favors the extraction of highly confident peptide CSD readings.

Moreover, the extraction scheme was specifically devised to estimate CSDs solely from MS1 intensities, thus avoiding biases that might result from precursor ion selection during MS/MS. Consequently, even though 1+ precursor ions were excluded from MS/MS analysis, the extraction scheme still detects 1+ charge states provided at least one other charge state is identified through MS/MS.

We analyzed a total of 326 raw LC–MS/MS files which resulted in CSD readings extracted for a total of 261 667 unique peptides (Table 1). The resulting CSD dataset has a similar distribution of peptides to those typically identified using MS/MS, with masses ranging from 700 to 4600 Da and varying numbers of basic sites (Figure 2).

**Table 1:** Overview of Peptide CSD Dataset^a^

	data source			
	ours	Confetti	Meier et al.	total
LC–MS/MS runs	20	18	288 (39*)	326 (77*)
Total peptides	264 259	166 543	416 306	847 108
Unique peptides	41 594	80 402	183 340	261 667
Varied parameters	gradient length, voltage, flow rate	protease	organism, protease	
MS instrument	Orbitrap	Orbitrap	timsTOF	

a Breakdown of extracted CSDs from each of the three data sources (ours, Confetti, Meier et al.). Asterisk (*) indicates the number of LC–MS/MS runs after aggregation over fractionations.

### Measuring Error between CSD Readings

Error between CSD readings of the same peptide was measured through the total variation. The total variation between two distributions (*p*_1_, ..., *p*_*n*_) and (*q*_1_, ..., *q*_*n*_) is given by the sum of absolute differences between probabilities divided by 2:
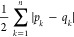
1Total variation ranges from 0 to 1, signifying equivalent or disjointed distributions, respectively.

### Correcting Experimental Batch Effects

To better assess CSD errors across pairs of runs, we applied a one-parameter batch correction (Figures S1 and S2). Peptide CSDs were transformed according to the following scheme. For each charge state *k*^+^, we scaled its probability *p*_*k*^+^_ by a factor of γ^*k*^, for some global batch parameter γ > 0. The scaled probabilities were then renormalized to sum to one, forming the transformed CSD. In other words, our batch correction maps peptide CSDs of the form (*p*_1^+^_, ..., *p*_5^+^_) to
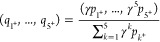
2This transformation can be interpreted as additive shifts in the energy scale (i.e., log probability scale), in which one run has log γ higher energy differences between consecutive charge states than the other run. We selected this transformation as we observed multiplicative-like batch effects. Namely, when comparing across pairs of runs, we observed that the log-odds between consecutive charge state probabilities were roughly offset by a fixed amount. That is, we observed that, typically,
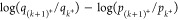
3was roughly the same across all peptides and all charge states *k*^+^ and (*k* + 1)^+^ in a given pair of runs (Figure S2a,c inset). Our batch correction can be derived through assuming the above expression is equal to some global constant (namely, log γ).

Errors across pairs of runs were measured before and after applying a batch correction (Figure S1). The batch correction parameter was chosen to minimize the total error, calculated as the sum of the total variation in the CSD readings across all peptides shared between both runs. We found this batch correction to be reasonably effective, given that it only depends on one parameter.

### Calculating and Analyzing Effective Basicity Scores

To analyze the effect of sequence on charging (see results; Figure 3), we used the coefficients of a logistic regression as a measure of feature impact. Specifically, for each run, we performed four separate regression analyses, each one using a subset of peptides across consecutive charge states *k*^+^ and (*k* + 1)^+^ for some *k* from 2 to 4 (“charging region”). As the dependent (target) variable of the regression we used the quantity *p*_(*k*+1)^+^_/(*p*_*k*^+^_ + *p*_(*k*+1)^+^_), which can be seen as the conditional probability of exhibiting charge state (*k* + 1)^+^ relative to *k*^+^. As the independent (input) variables, we used 20 numerical variables, one for each of the 20 amino acid counts, and 20 binary variables to denote the identity of the N-terminal amino acid. The regression is given by the equation
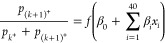
4where *f*(*x*) = 1/(1 + e^–*x*^) is the standard logistic link function, and *x*_*i*_’s are the regression variables. The above can be viewed as a linear relationship when written in terms of the log-odds of *p*_(k+1)^+^_ to *p*_*k*^+^_, that is,
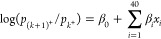
5In other words, the coefficient β_*i*_, which we term the “effective basicity score” for variable *i*, can be interpreted as an additive contribution in the log-odds scale for charging across charge states (*k* + 1)^+^ and *k*^+^. The logistic regression was performed using the pyGAM library,^37^ with binary cross entropy as the loss function and mild L2 regularization (λ = 0.01).

To compare across region-run pairs (Figure 3c), we calculated the Pearson correlation coefficient of the effective basicity scores for the 17 nonbasic amino acids. Only our LC–MS/MS runs with sufficiently many data points in all four charging regions were considered; specifically, each charging region needed to contain >100 peptides that exhibited nonzero probabilities on both charge states in question. The resulting LC–MS/MS runs, which were used in Figure 3c, are the 12 runs from the April 2021 experiment.

To calculate mass-adjusted effective basicity scores for each region-run pair (Figure 3d), we subtracted from the overall effective basicity scores the portion that could be explained by mass. Specifically, the mass-adjusted effective basicity scores were derived as the residuals of a linear regression between the effective basicity scores and the masses of the 17 nonbasic amino acids plus an intercept term. The linear regression used a Huber loss^38^ with parameter δ = 0.01. We selected the Huber loss, which is equal to the L1 loss for large values (>δ) and the L2 loss for small values (<δ), to ensure that amino acids with effective basicity scores significantly different from the mass trend were not overly penalized and to guarantee uniqueness of the coefficients.

## Results and Discussion

### Overview of Peptide CSD Dataset

To facilitate a machine learning approach, we developed an extraction scheme to extract CSD readings from MS1 scans (see Methods). In each LC–MS/MS run, a single CSD reading was assigned to each MS2-identified peptide by averaging CSD readings across the peptide’s elution.

To cover a wide range of experimental settings, we applied our extraction scheme to 326 positive-ion mode LC–MS/MS runs acquired from three sources: our own, Confetti,^31^ and Meier et al.^32^ These data sources differ in their choice of experimental parameters, protease, organism, and type of mass spectrometry instrument (Orbitrap^39^ and timsTOF^40,41^). The resulting dataset contained CSD readings of 261 667 unique peptides (Table 1).

In order to confirm the reproducibility of the extracted CSD readings, we compared CSDs from various LC–MS/MS runs and assessed potential sources of errors. We found that CSD readings of the same peptide were generally consistent across different runs (Figure S1), and especially consistent among experimental replicates (∼3.8% error Orbitrap, ∼5.2% error timsTOF). After applying a one-parameter batch correction (see Methods), errors across replicates dropped slightly (∼3.5% error, Orbitrap; ∼4.7% timsTOF). (We remark, however, that batch correction was not used in any downstream analysis; instead, LC–MS/MS runs were analyzed separately in order to avoid introducing biases.) Furthermore, the datasets from timsTOF instruments had slightly higher errors (Figure S1), which may be due to the extraction scheme being insufficiently optimized to that technology (as it does not employ ion mobility information). Lastly, one potential source of error is the mass spectrometer’s intensity threshold. Since charge states with intensities that fall below the threshold do not appear in the mass spectrum, this may introduce biases in CSD readings for less abundant peptides, leading to “rounding” artifacts in mean charge. We verified that the intensity threshold only accounts for <2% error for most CSD readings, as our extraction scheme’s filtering steps naturally favor the extraction of high intensity peptides. Together, these results indicate that peptide CSDs are highly consistent across replicates, and demonstrate the robustness of the extraction scheme.

**Figure 1:**
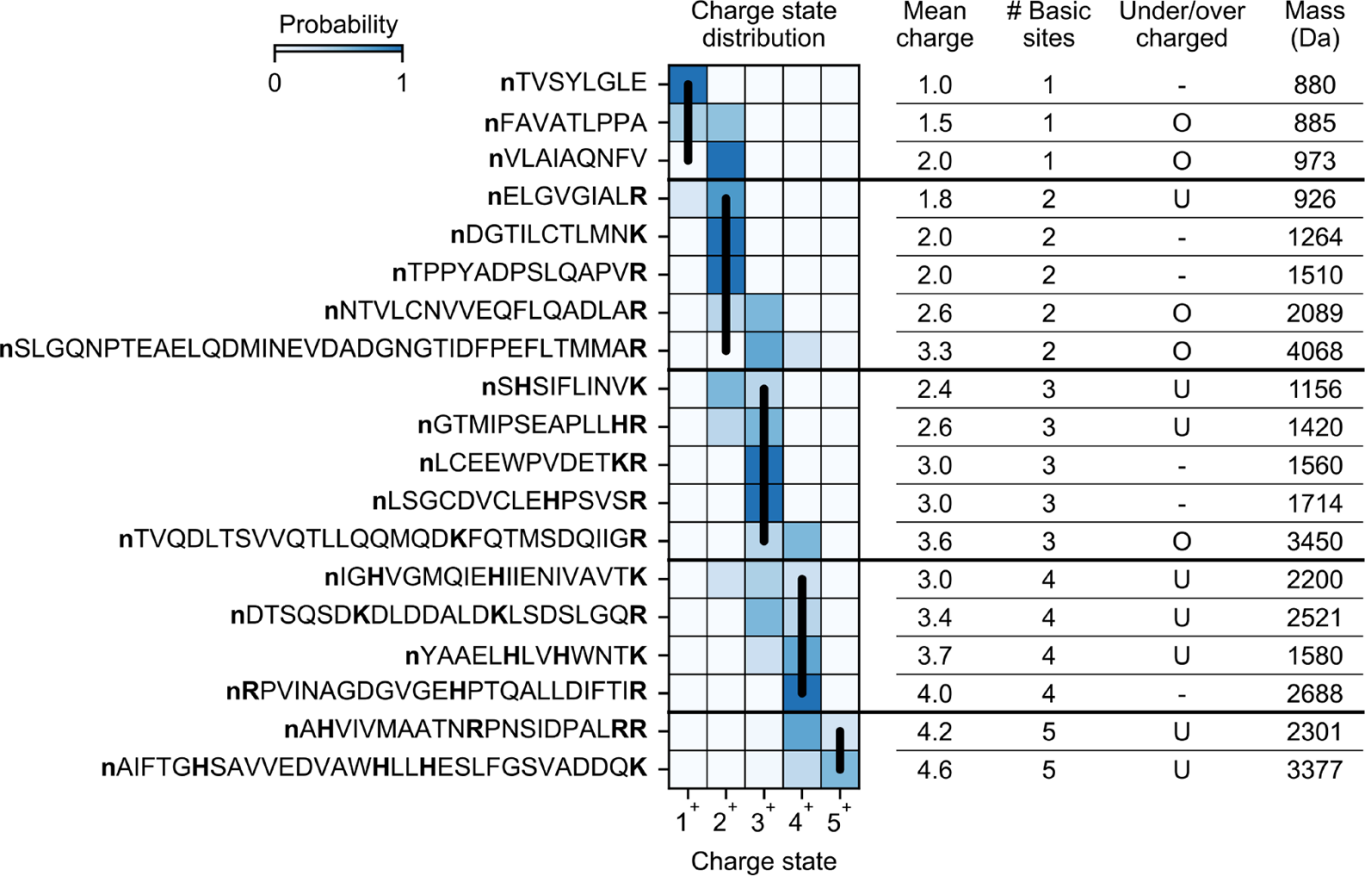
CSDs of select peptides. Extracted CSDs and properties of select peptides from one of our HeLa trypsin runs (2.5 kV ESI voltage, 160 min gradient length, 400 nL/min flow rate). Basic sites are bolded. Vertical black lines denote the charge state equal to basic site count. U = undercharged (mean charge < basic site count). O = overcharged (mean charge > basic site count).

Peptides in our dataset exhibited a variety of CSDs (select peptides shown in Figure 1). It is known that the basic site count serves as a rough estimate for the charge a peptide receives in positive-ion mode ESI.^42^ Indeed, 51% of the peptides in the dataset have a CSD concentrated solely on the charge state equal to the basic site count. On the other hand, 40% of peptides exhibit undercharging (mean charge less than basic site count), and 9% exhibit overcharging (mean charge greater than basic site count). In downstream analysis, we explore factors that explain why some peptides receive fewer charges or more charges than their basic site count.

### Under- and Overcharged Peptides Exhibit Different Dependence on Mass and Number of Basic Sites

**Figure 2:**
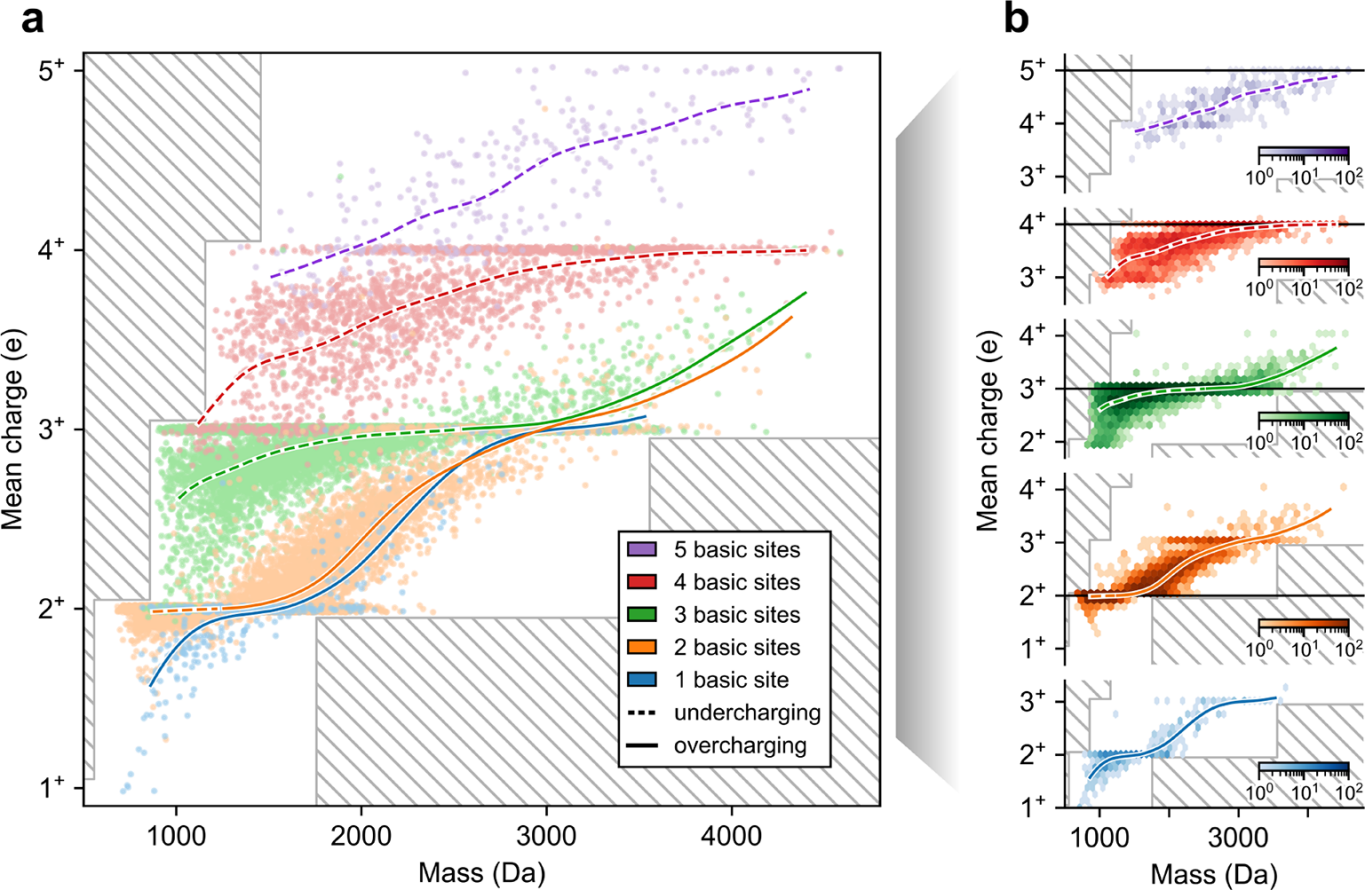
Visualizing CSD dataset versus mass. Plots of mean charge versus mass for peptides from a representative run (our HeLa trypsin run; 2.5 kV ESI voltage, 160 min gradient length, 400 nL/min flow rate), colored by number of basic sites. Data points are shown as (a) a scatter plot with jittering in the *y*-axis, uniformly chosen from −0.02 to 0.02, and (b) 2D hexagonal binning plots separated by basic site count. Colored curves show spline-interpolation of mean charge versus mass, performed with 20 cubic-splines, a smoothing parameter of 5, and a monotonicity constraint. Curves are dashed when the interpolated mean charge is less than the basic site count (undercharging) and solid otherwise (overcharging). Hatched regions correspond to unobservable mean charge due to *m*/*z* cutoff.

Grouping peptides by their basic site count, we observed that mean charge increases with mass and exhibits transitions from under- to overcharging in all LC–MS/MS runs (representative runs shown in Figures 2, S3, and S4). For instance, in one of our HeLa runs, among peptides with three basic sites and mass less than 2600 Da, 98% exhibited charges of 3^+^ or lower (undercharging, Figure 2b). In contrast, among peptides with three basic sites and a mass greater than 2600 Da, 94% exhibited charges 3^+^ or higher (overcharging, Figure 2b). We confirmed that this transition phenomenon cannot be explained by the mass spectrometer’s *m*/*z* cutoff, since the presence of a charge state recedes well before reaching the *m*/*z* cutoff. Moreover, the phenomenon is present in runs performed with other proteases (Figure S3) and with the timsTOF instrument (Figure S4), suggesting it is independent of the peptide distribution or the choice of mass spectrometer instrument.

Prior studies on select protein complexes^43−45^ and synthetic peptides^46^ have also observed correlations between size and charging. Moreover, the relationship is consistent with proposed ionization theories^27^ as larger analytes would be enclosed in larger, more highly charged droplets,^20^ may have higher solvent-exposed surface area,^44,45^ and may have more functional groups that can stabilize charge through intramolecular solvation.^47−49^ Our findings suggest that this correlation holds universally for peptides and provide quantitative dependencies between the number of basic sites, mass, and mean charge.

One striking observation is that in the overcharging regime, the number of basic sites has little effect on the mean charge trend as a function of mass (Figure 2a, solid lines). In contrast, in the undercharging regime, the number of basic sites has a significant effect on the mean charge trend (Figure 2a, dashed lines). Thus, in the overcharging (but not in the undercharging) regime, mass takes precedence over the number of basic sites. For instance, among peptides of mass 1800 Da, those with 1 or 2 basic sites exhibit, on average, similar mean charges of 2.1 and 2.2, respectively (overcharging), whereas those with 3, 4, or 5 basic sites exhibit, on average, distinct mean charges of 2.9, 3.4, and 4.0, respectively (undercharging). One potential explanation for this phenomenon is that overcharged peptides, having high mass relative to their basic site count, contain enough backbone carbonyls for solvating excess protons.^49^ As such, additional basic sites may not have a strong effect on overcharging. There may be other potential explanations for this phenomenon, including causes arising outside of ESI such as the efficiency of transporting ions into the mass analyzer. Regardless, the striking contrast in basic site count dependence suggests that there are differences in the underlying processes that govern under- and overcharging of peptides.

These findings also demonstrate the potential benefits that CSDs can offer peptide identification. Aside from the minority of peptides that exhibit overcharging, peptides sharing the same mass but having a different number of basic sites generally exhibit different CSDs (Figure S5), allowing them to be distinguished based on the MS1 scan. For example, among peptides with mass 1600 ± 25 Da, 99% of those with two basic sites have mean charge of <2.5, while 98% of those with three basic sites have mean charge of >2.5. As such, when one is searching for potential peptide candidates for a given collection of ion peaks, the observed mass and CSD can be used to infer the peptide’s basic site count. This example showcases a preliminary use-case for peptide CSDs, and suggests that incorporating more sequence-dependent insights may provide further identification opportunities.

### Distinct Sequence Determinants Underlie Peptide Ionization in the Under- and Overcharging Regimes

A peptide’s ionization depends on many factors, including Coulombic forces,^50,51^ its gas-phase conformation,^48,52^ its protonatable locations,^53,54^ solvent acidity/basicity,^55^ and intramolecular forces.^47−49^

To provide further insights into the sequence factors that influence peptide ionization, we analyzed the sequence determinants of CSDs within the under- and overcharging regimes.

**Figure 3:**
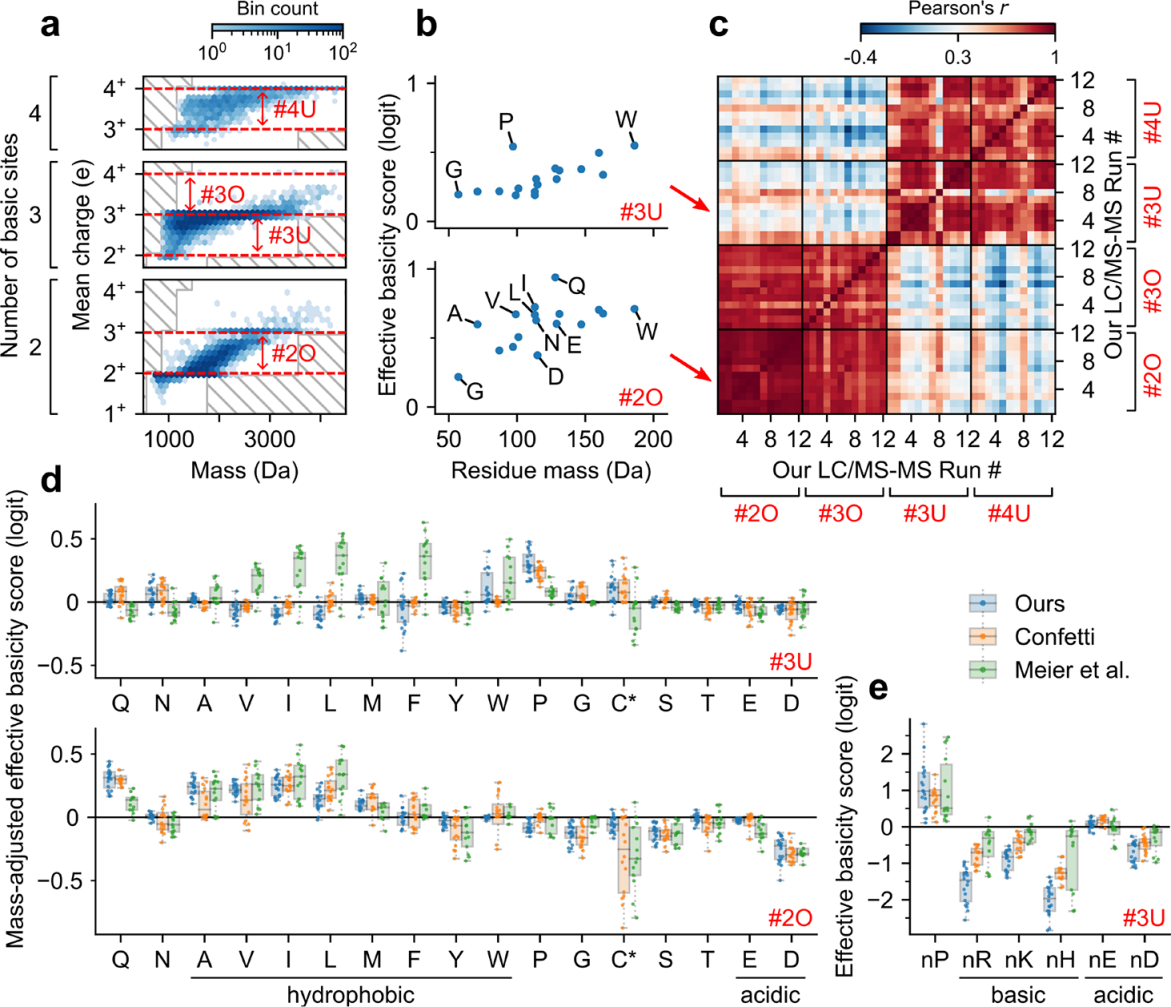
CSD sequence determinants for under- and overcharged peptides. (a) Schematic representation of the four peptide charging regions of interest, determined by the number of basic sites and pair of consecutive charge states (see text). (b) Scatter plots of amino acid effective basicity scores (see text) versus mass for two representative charging regions (top, region #3U; bottom, region #2O). Panels a and b use our HeLa trypsin run with 2.5 kV ESI voltage, 160 min gradient length, and 400 nL/min flow rate. (c) Heatmap showing correlations between amino acid effective basicity scores among all pairs of region-run combinations for our runs with sufficiently many data points (see Methods). Along both axes, all 12 runs are aligned four times, once for each of the four regions. (d) Box plot of estimated mass-adjusted effective basicity scores (given by residual effective basicity scores after subtracting trend in mass; see Methods) across runs with sufficiently many data points, separated by data source and charging region. Runs from Meier et al. were aggregated across fractionations (average taken for duplicate readings). C* denotes carbamidomethyl-cysteine in our and Meier et al.’s runs, and *N*-ethylmaleimide modified cysteine in Confetti’s runs. (e) Box plot of effective basicity scores for the N-terminal identity of select amino acids (region #3U). Box plot elements: centerline, median; boxplot limits, upper and lower quartiles; whiskers, 1.5× interquartile range; points, in whisker range with jitters (outliers not shown).

For the analysis, we considered the 20 amino acid counts and the identity of the N-terminal amino acid as the sequence features of interest. To account for possible dependence on the number of basic sites or charge states, we performed a separate analysis for each of four regions, labeled #2O, #3O, #3U, and #4U (O, overcharged; U, undercharged; Figure 3a). Region #2O consists of peptides with two basic sites and considers charging across 2^+^ and 3^+^. We similarly define region #3O (three basic sites, charging across 3^+^ and 4^+^), region #3U (three basic sites, charging across 2^+^ to 3^+^), and region #4U (four basic sites, charging across 3^+^ and 4^+^). For each of our 12 LC–MS/MS runs (with sufficiently many data points, see Methods) and each of the four regions, we estimated a feature’s impact on charging using an effective basicity score (Figure 3b), derived from the coefficients of a logistic regression (positive effective basicity corresponding to higher charging; see Methods). Interestingly, clustering the region-run pairs by their effective basicity scores, we identified two distinct clusters (Figure 3c): there was strong agreement only between regions #2O and #3O, and between regions #3U and #4U. This result suggests that under- and overcharging are influenced by different sequence features and that these sequence features do not depend strongly on the number of basic sites.

To understand the sequence determinants of overcharging, we examined some of the more prominent effective basicity scores for region 2O across all runs. We observed that glutamine (Q) and aspartic acid (D) have one of the highest and lowest mass-adjusted effective basicity scores, respectively (Figure 3d, bottom; Figure S6a, bottom). These observations are consistent with previous work: glutamine (Q) has been reported to have high gas-phase basicity due to its amide group^53,56^ and can be a potential charge carrier during ESI;^53^ aspartic acid (D) has been observed to form salt bridges with basic sites in molecular dynamics simulations.^57^ Interestingly, despite having the same functional group, glutamine (Q) displays a notably higher effective basicity score than asparagine (N); similarly, glutamic acid (E) displays a notably higher effective basicity score than aspartic acid (D) (Figure 3b,d). Both pairs of amino acids differ only in side-chain length, suggesting that the differences in effective basicity scores are associated with differences in conformational entropy.^58^ Lastly, we observed that amino acids with nonaromatic hydrocarbon side chains (alanine (A), valine (V), isoleucine (I), and leucine (L)) have high mass-adjusted effective basicity scores. As nonpolar moieties increase a peptide’s affinity for the droplet-air interface,^59^ this finding suggests that peptides positioned closer to the droplet surface may exhibit a stronger overcharging response, which is consistent with the ion evaporation and chain ejection models of ion formation.^57^

Next, to understand the impact of sequence features on undercharging, we examined some of the more prominent effective basicity scores for region 3U across all runs. We observed that the presence of an arginine (R), lysine (K), or histidine (H) at the N-terminal greatly reduces charging (Figure 3e; Figure S6b, top). This suggests that undercharging occurs due to Coulombic repulsion between the N-terminus and an N-terminal basic side chain. In particular, this effect can be observed in sequential isomers TNSTFNQVVLKR and RTNSTFNQVVLK, which have identical sequences apart from an arginine at the C- or N-terminal. In the four runs that contain CSD readings for both peptides, the former peptide has a CSD of (*p*_2^+^_, *p*_3^+^_) = (4%, 96%) ± 7% (mean ± sd; not batch corrected), while the latter, with an N-terminal arginine, has a CSD of (*p*_2^+^_, *p*_3^+^_) = (44%, 56%) ± 6%. In addition to these Coulombic effects, we observed that proline (P) has a notably high effective basicity score (Figure 3d, top; Figure S6a, top), especially if it is located at the N-terminus (Figure 3e; Figure S6b). We speculate that internal prolines may reduce undercharging by introducing a kink in the peptide chain,^60^ thereby promoting charge solvation^49^ and protecting from Coulombic repulsion. Lastly, the timsTOF runs (from Meier et al.) demonstrated higher mass-adjusted effective basicity scores for strongly hydrophobic amino acids (valine (V), isoleucine (I), leucine (L), and phenylalanine (F)) than did the Orbitrap runs (Figure 3d, top).

This analysis has some limitations. First, CSDs are influenced by all factors that occur in and downstream of ESI, including ionization efficiency, transport efficiency (from ESI to MS detection),^59^ and possible interactions within the drift tube.^61^ Due to these factors, it can be challenging to attribute the effects identified in our analysis to any one of these processes. We partly address this issue by including runs from both the Orbitrap and timsTOF mass spectrometers. Moreover, our analysis can only establish correlations, not causal relationships, in the dataset. Further studies are necessary to fully establish the mechanisms underlying the findings that we have identified.

In conclusion, our analysis demonstrates that the under- and overcharging regimes are influenced by distinct sequence determinants. These sequence determinants are largely consistent across different LC–MS/MS runs and align with previously documented effects. Moreover, our findings indicate that peptide CSDs depend on many factors beyond mass and basic site count, including both the composition and position of the constituent amino acids. Collectively, these results underscore the value of CSDs in providing information about peptide sequences and suggest that CSDs may complement other measured analyte properties for proteomics identification.

### CSD Variations Offer Opportunities for Identification

It is well-known that certain LC–MS/MS experimental parameters change the overall degree of charging experienced by analytes.^3,42,55,59,62^ Indeed, in our own experiments, we observed that increasing flow rate and gradient length generally resulted in higher overall charging (whereas ESI voltage had a minimal effect; Figure S7). While such experimental variations may be perceived as undesirable, they can be potentially harnessed to improve peptide identification. Specifically, peptides with similar CSDs may exhibit more distinct CSDs under different charging conditions, allowing for easier separation.

To illustrate this concept, consider the two peptides ASGQAFELILSPR and ACANPAAGSVILLENLR. In the low flow rate runs (200 nL/min), these peptides exhibited CSDs of (*p*_2^+^_, *p*_3^+^_) = (96%, 4%) and (88%, 12%), respectively, a difference of 8%. In the high flow rate runs (800 nL/min), these peptides exhibited CSDs of (*p*_2^+^_, *p*_3^+^_) = (90%, 10%) and (74%, 26%), respectively, a difference of 16%. In other words, increasing the flow rate resulted in a 2-fold increase in the difference between these two CSDs. In fact, this example occurs throughout our dataset (Figure S2a,b). For instance, among peptides with mean charge between 2 and 2.15 in the low flow rate run, more than half of the pairwise differences (59%) increased by 2-fold or more when flow rate increased (Figure S2a). Similarly, among peptides with mean charge between 2.85 and 3 in the high flow rate run, more than half of the pairwise differences (52%) increased by 2-fold or more when flow rate decreased. Lastly, CSD variations across other pairs of runs exhibited similar trends (Figure S2b). Together, these findings highlight how varying experimental parameters can magnify differences in CSDs.

Instead of varying experimental parameters across runs, one can also imagine inducing variations in charging within a single run, possibly on a scan-to-scan basis. In fact, we observed that in many runs mild scan-to-scan charging fluctuations already exist. While the reasons for these fluctuations are not clear, our findings indicate that they are not due to noise and have potential for improving identification (Supplementary Text 1).

In summary, these findings suggest that varying charging conditions across runs and within a single run may induce predictable modulations in CSDs, enabling potential applications in peptide identification.

## Conclusion

This work identified general patterns underlying peptide ionization through constructing a large-scale CSD dataset (>800 000 peptides) and employing a machine learning-based analysis. We found that the interplay of mass and basic site count largely determines under- and overcharging with further fine-tuning based on additional sequence-dependent features. Notably, our analysis indicates that basic site count does not strongly influence mean charge in the overcharging regime, that Q, A, V, I, and L are correlated with higher charging for overcharged peptides, and that N-terminal basic sites are correlated with lower charging for undercharged peptides. Furthermore, we demonstrated that peptides can exhibit a differential response to experimental conditions. Together, these results highlight potentially useful information contained in peptide CSDs that may benefit bottom-up proteomics and that is not currently utilized in state-of-the-art identification and quantification pipelines.^34,63^ In addition to data-dependent acquisition (DDA),^64^ our findings may especially be useful for data-independent acquisition (DIA)^65^ and MS1-only^66^ approaches, where there are no fragmentation spectra dominated by one peptide. In conclusion, our data-driven study of peptide CSDs has yielded informative results, potentially shedding more light on ESI mechanisms and offering applications to mass spectrometry-based proteomics.

## Data Availability

Raw files and MaxQuant analyses for our LC–MS/MS runs are available at the MassIVE data repository with ID MSV000091473. The CSD dataset generated and analyzed in this study is available at figshare.^67^

## Supporting Information Available

List of LC–MS/MS runs (XLSX)
Supplementary figures illustrating reproducibility of the CSD extraction scheme, overviews of extracted CSDs, and additional analyses; supplementary text summarizing findings on scan-to-scan CSD fluctuations; supplementary text with the full details of the CSD extraction scheme (PDF)

## Notes

**Code Availability.** Extraction scheme and figure generating code are available on GitHub (https://github.com/regev-lab/extract-csd).
