# Supplementary material for: Uncovering Distinct Peptide Charging Behaviors in Electrospray Ionization Mass Spectrometry Using a Large-Scale Dataset
Source: J Am Soc Mass Spectrom. 2023 Dec 14;35(1):90–9. doi: 10.1021/jasms.3c00325 (PMC10767741; doi:10.1021/jasms.3c00325)
Supplement: Supplementary file 2 — js3c00325_si_002.pdf [file js3c00325_si_002.pdf]

Supporting Information

Uncovering Distinct Peptide Charging Behaviors in Electrospray Ionization Mass Spectrometry Using a Large-Scale Dataset

Allyn M. Xu[1], Lauren C. Tang[2], Marko Jovanovic[2] & Oded Regev[3*]

The supporting information includes 9 figures, and 2 notes.

[1]Department of Mathematics, Courant Institute of Mathematical Sciences, New York University, NY 10012, USA.
[2]Department of Biological Sciences, Columbia University, New York, NY 10027, USA.
[3]Computer Science Department, Courant Institute of Mathematical Sciences, New York University, NY 10012, USA.
[*]e-mail: regev@cims.nyu.edu

Supplementary Data

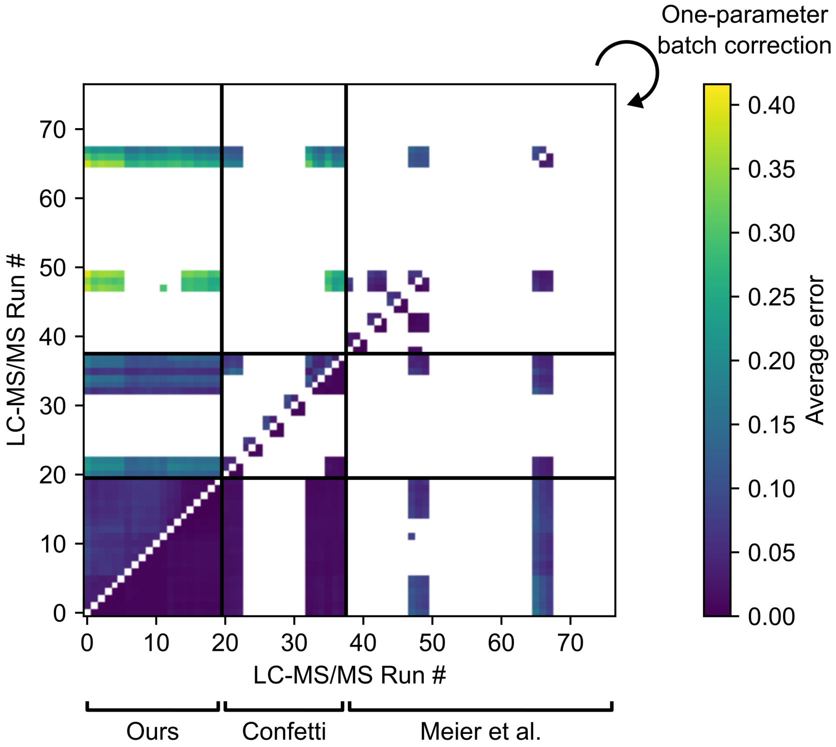

Figure S1. **Error across runs before and after batch correction.** Color-coded matrix of average CSD error across pairs of runs (with $\geq$ 200 shared peptides) measured before (upper left) and after (lower right) applying a one-parameter batch correction (see methods). Error is measured as average total variation (see methods) between CSD readings of shared peptides. Meier et al.'s runs were aggregated across fractionations (average taken for duplicate readings).

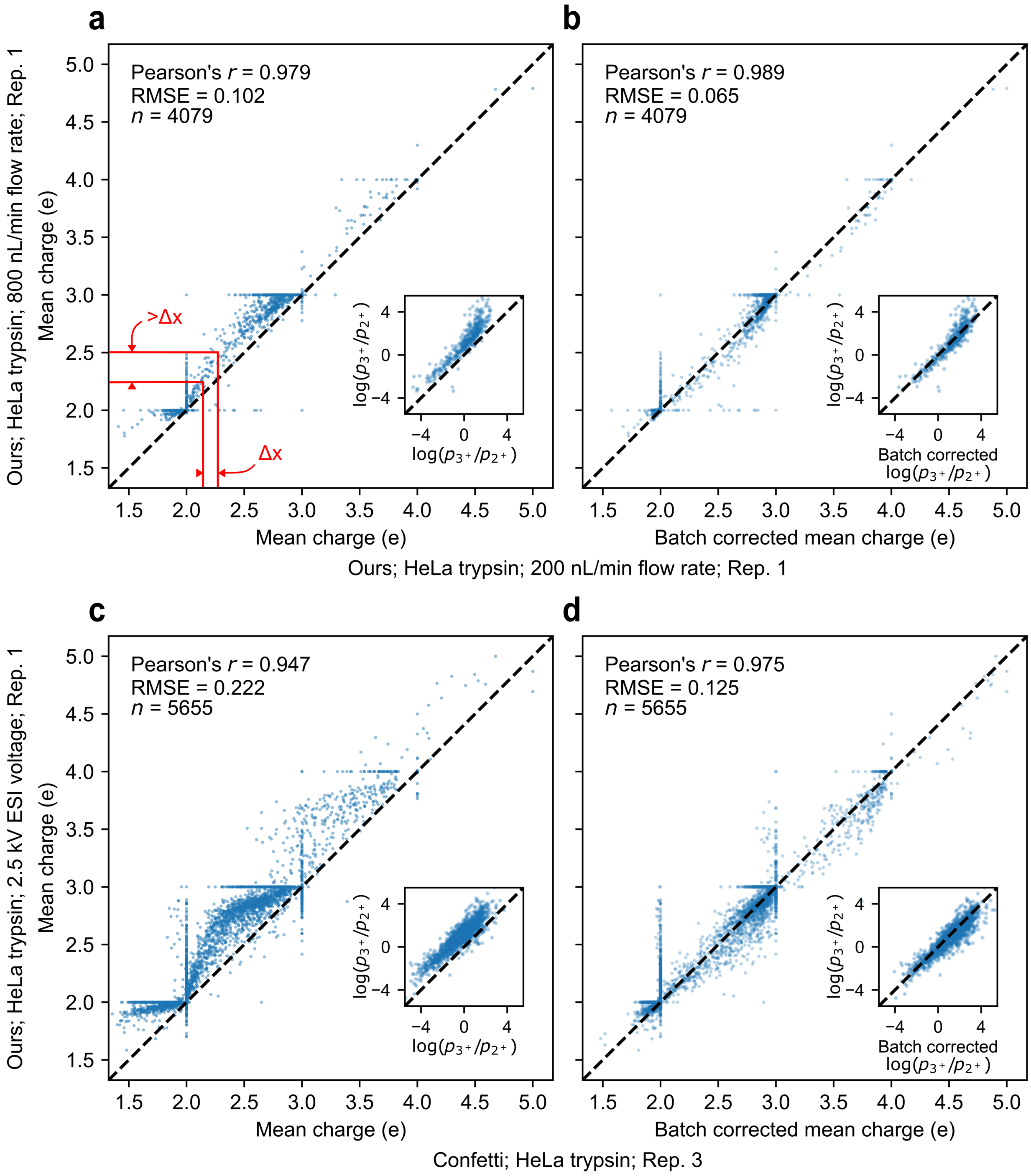

Figure S2. Examples of before and after batch correction. Comparison of mean charge readings of shared peptides between (a,b) our low and high flow rate runs, and (c,d) a Confetti run and our high voltage run. Mean charge is shown before (a,c) and after (b,d) applying a one-parameter batch correction (see methods). Insets show comparison of log odds between charge states 2^+ and 3^+ of shared peptides. Red lines and text in (a) illustrate the effect of experimental variation on mean charge differences.

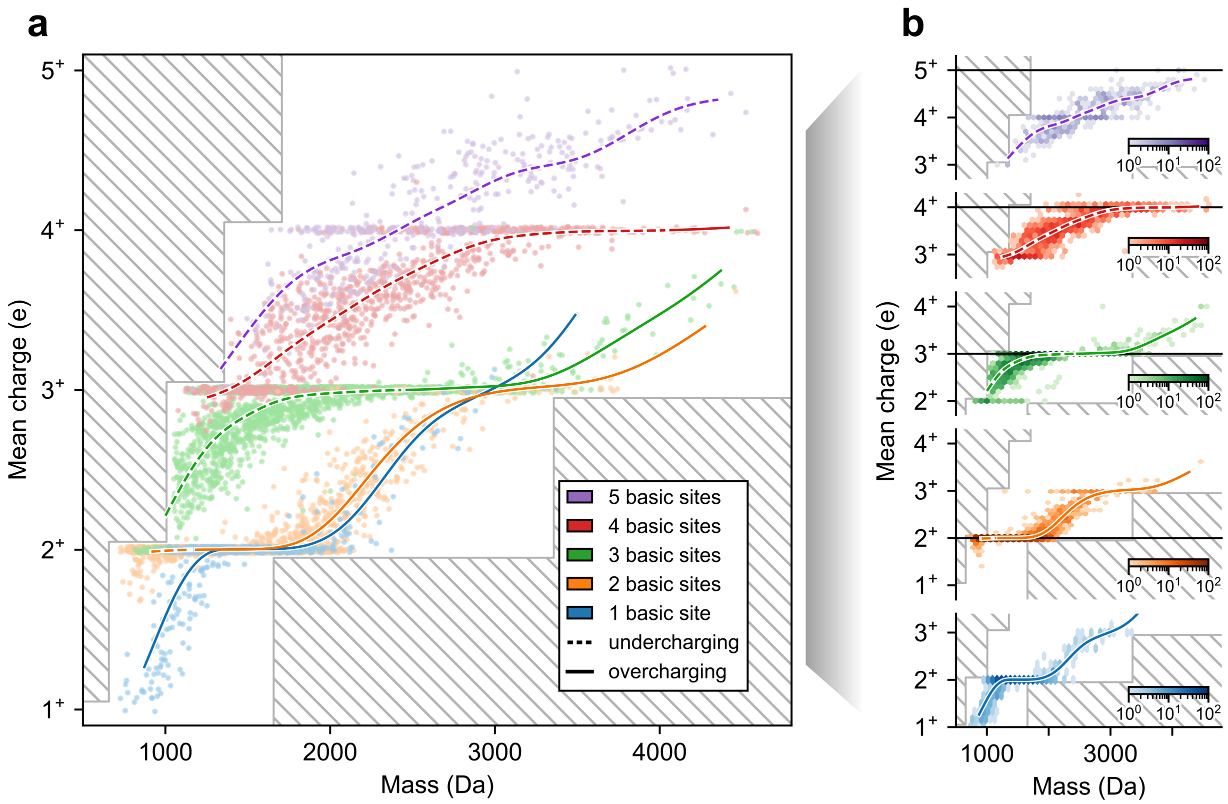

Figure S3. **Visualizing CSD dataset versus mass for a Confetti run.** Plots of mean charge versus mass for peptides from a representative run (Confetti; HeLa GluC) as in Figure 2(a–b).

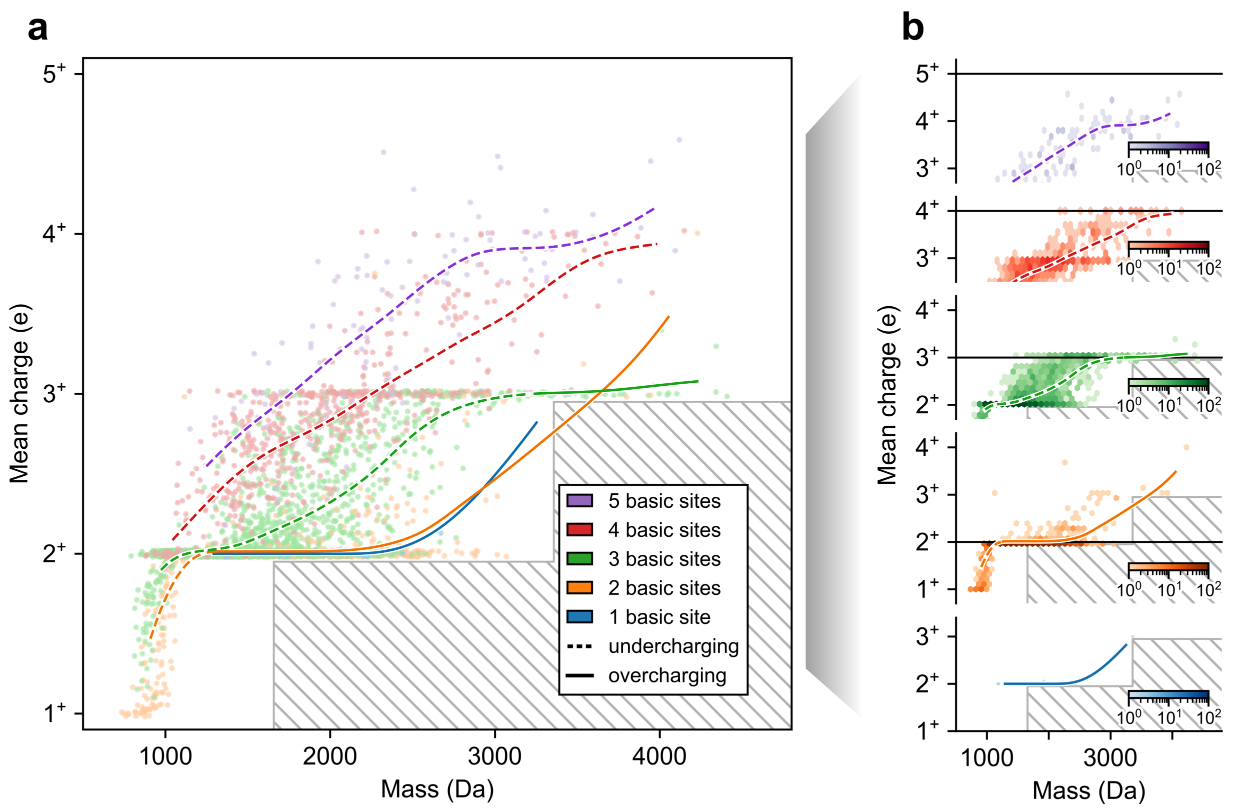

Figure S4. Visualizing CSD dataset versus mass for a run from Meier et al. Plots of mean charge versus mass for peptides from a representative run (Meier et al.; HeLa trypsin) as in Figure 2(a–b).

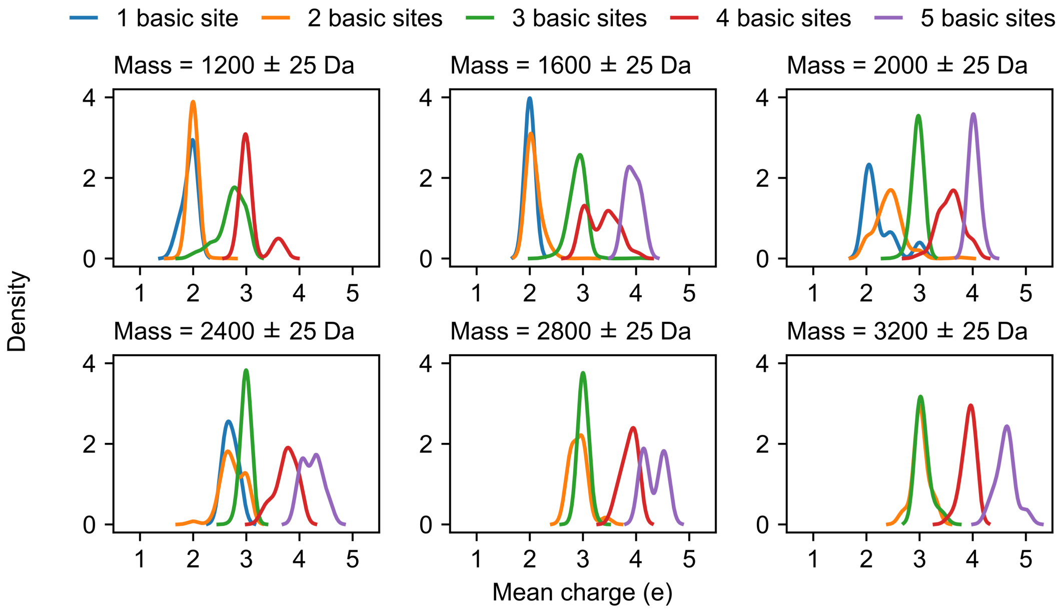

Figure S5. Mean charge for different basic site counts are generally disjoint. Distributions of peptide mean charge, separated by number of basic sites, shown along different slices of mass. Kernel density estimation is performed with a gaussian kernel (bandwidth = 0.1). Data shown is from our HeLa trypsin run with 2.5 kV ESI voltage, 160 min gradient length, 400 nL/min flow rate.

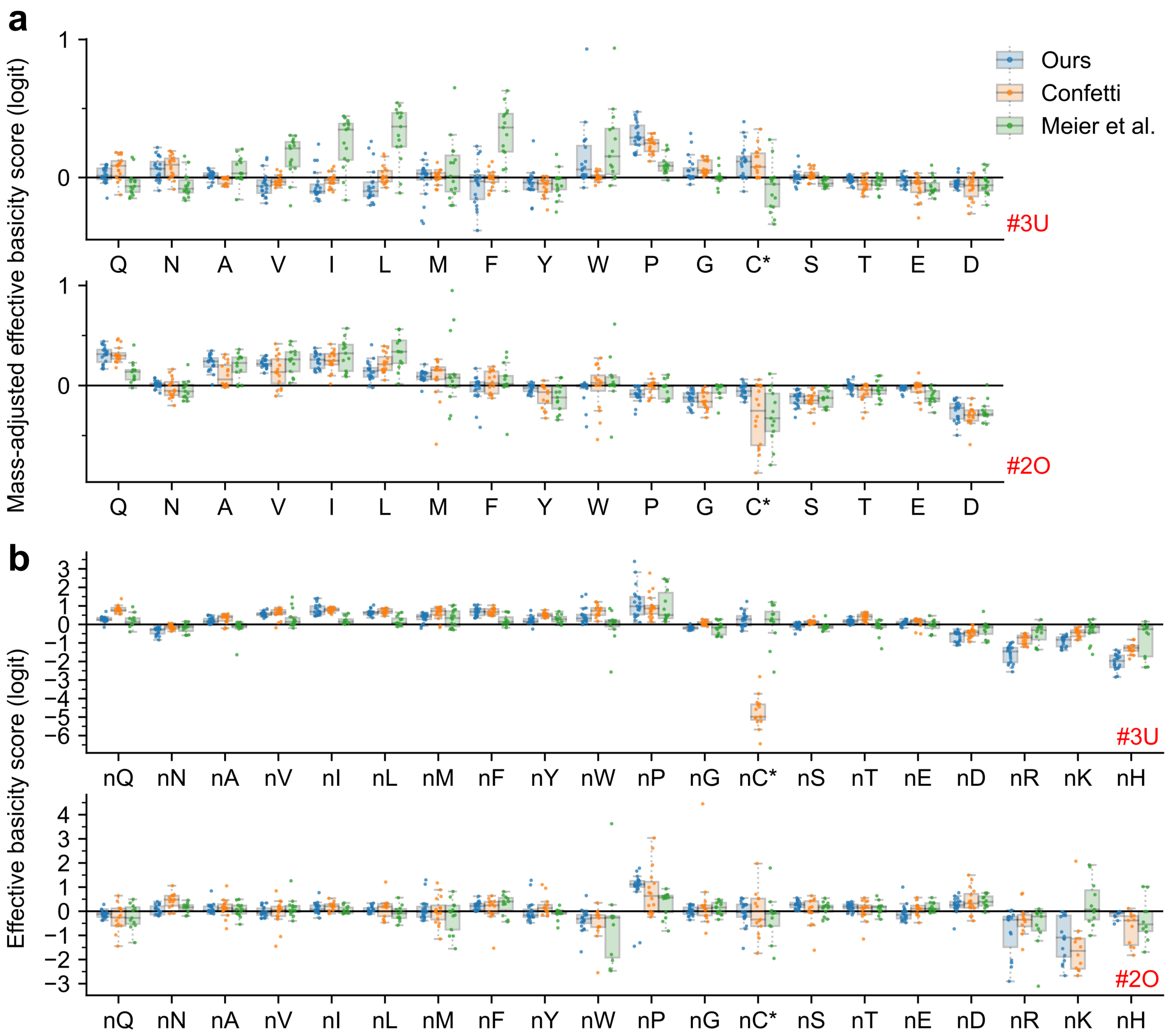

Figure S6. **Extended versions of Figure 3d,e.** Box plot (with outliers) of (a) estimated mass-adjusted effective basicity scores (given by residual effective basicity scores after subtracting trend in mass, see methods) for amino acids and (b) effective basicity scores for the identity of N-terminal amino acid across runs, separated by data source and charging region. C* denotes carbamidomethyl-cysteine in our and Meier et al.'s runs, and N-ethylmaleimide modified cysteine in Confetti's runs. Box plot elements: centerline, median; boxplot limits, upper and lower quartiles; whiskers, 1.5x interquartile range; points, all with jitters.

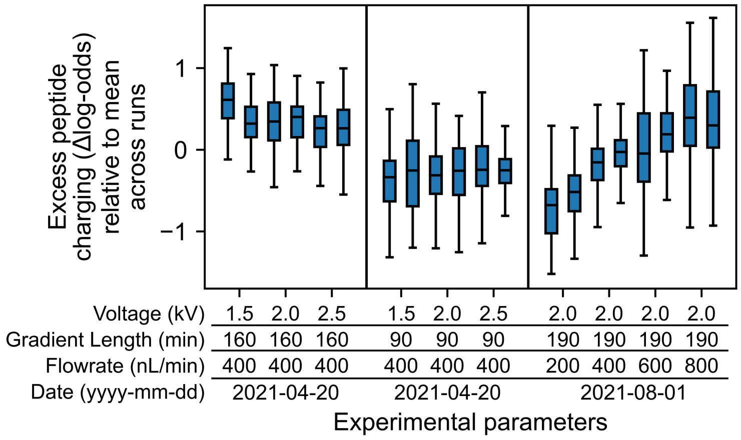

Figure S7. **Relative charging across runs.** Box plot showing distribution of excess charging across runs for 124 peptides shared between all our runs. Excess peptide charging is the difference between a peptide's mean log odds across its elution to the mean of that value across all runs. Log odds is defined as the log of the ratio of consecutive charge states. Box plot elements: centerline, median; boxplot limits, upper and lower quartiles; whiskers, 1.5x interquartile range; points, none (outliers not shown).

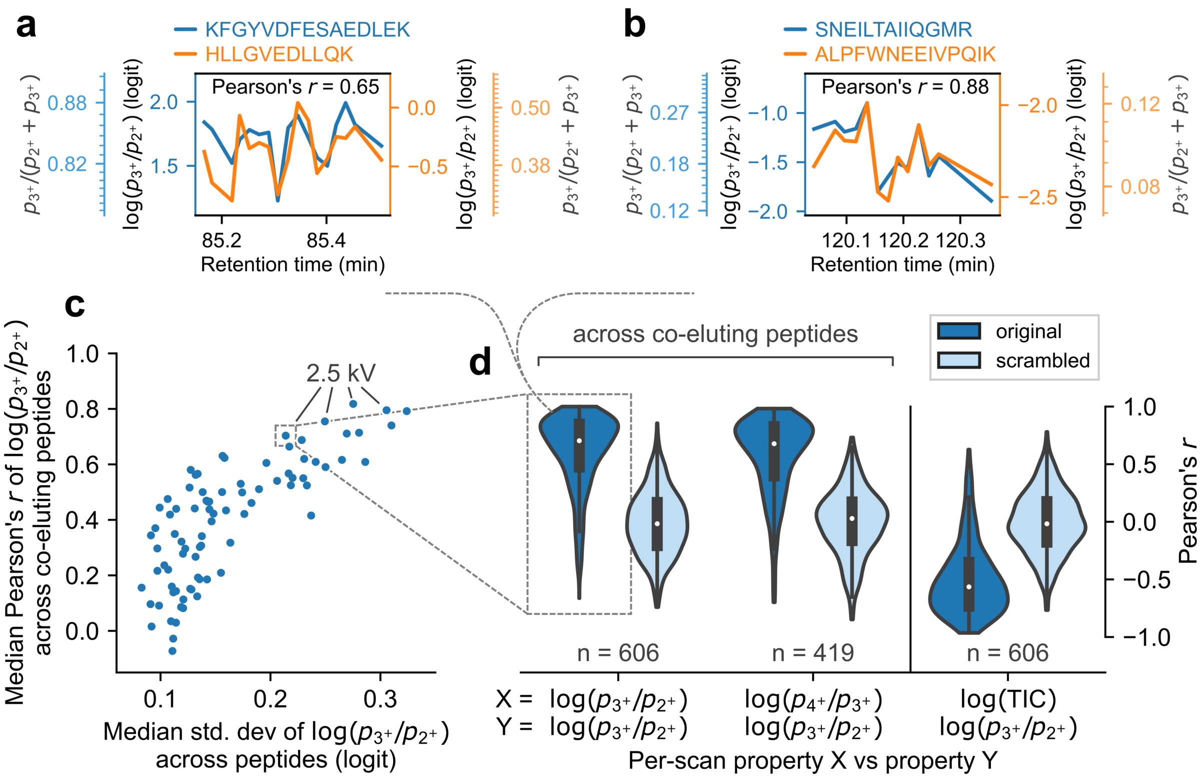

Figure S8. **Per-scan CSD fluctuations and effect of experimental parameters on charging.** (a,b) Fluctuations in CSD readings of two representative pairs of co-eluting peptides across their shared scans. The y-axes show the log odds between charge states 3^+ and 2^+. The corresponding values of the (non-log) odds are shown in the outer y-axes for comparison. (c) Scatterplot, across runs, of median correlation between CSD fluctuations of co-eluting peptides (>10 shared scans) versus median standard deviation in CSD fluctuation. Runs were only included if they contained >15 co-eluting peptides. Our runs with 2.5 kV ESI voltage are labeled. CSD fluctuations are measured as the log odds between charge state 3^+ and 2^+. (d) Violin plot showing correlations between log odds of co-eluting peptide CSDs (left and middle columns), and between log odds of peptide CSDs and total ion current (right column) for our HeLa trypsin run (2.5 kV ESI voltage, 160 min gradient length, 400 nL/min flow rate). Correlations taken after scrambling CSD readings across co-elution are shown as controls. Violin plot elements identical to previously defined box plot elements.

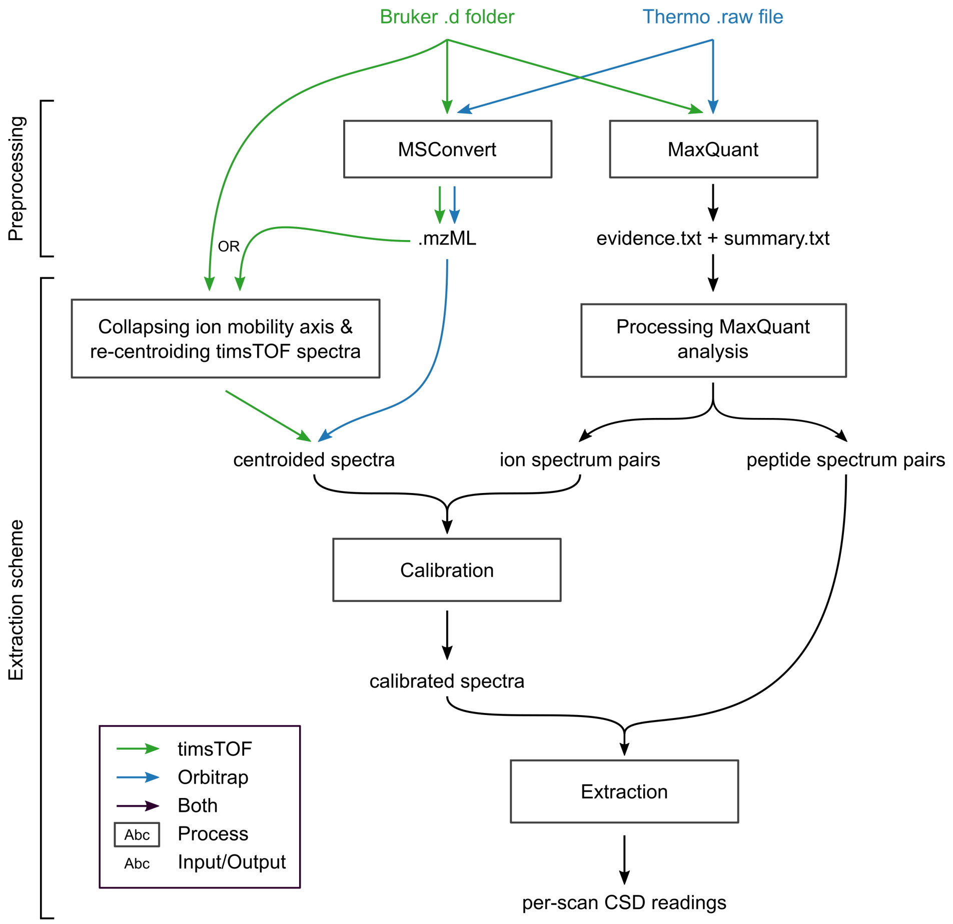

Figure S9. Infographic for the extraction scheme. Flowchart outlining steps taken to process raw LC-MS/MS files to obtain per-scan CSD readings.

Supplementary Text 1: CSD variations across scans offer opportunities for identification

In the main text, we assigned a single CSD reading to each peptide, which was obtained through averaging per-scan CSD readings across the peptide's elution. In this section, we report our findings on how CSD readings for the same peptide differ across scans.

We observed that CSDs exhibited mild scan-to-scan fluctuations, ranging from $\sim$1–5% depending on the LC-MS/MS run. To verify whether these CSD fluctuations were due to noise or due to scan-to-scan differences in overall charging, we compared the fluctuations of co-eluting peptides (Figure S8a,b). In most runs, we found that co-eluting peptides exhibited high correlation in their scan-to-scan charging, the only exceptions being runs that showed little to no fluctuations (Figure S8c,d). Moreover, a representative high-correlation run (one of our 2.5 kV runs) demonstrates that strong correlation is present not only on average but also for nearly all pairs of co-eluting peptides (Figure S8d, left). These correlations were also consistently observed regardless of the choice of charge states: comparing charging across 2^+ and 3^+ from one peptide with charging across 3^+ and 4^+ from another co-eluting peptide showed equally strong correlations (Figure S8d, center). In summary, these findings indicate that CSD fluctuations are largely attributable to variations in the overall degree of charging across scans.

Next, we sought to identify per-scan features that correlated with CSD fluctuations and can provide insights into their underlying cause. Among the features available from the LC-MS/MS files, we identified negative correlation (median Pearson's r across peptides $= -0.57$) between per-scan charging and total ion current (TIC), defined as the total intensity of all ion peaks in the scan (Figure S8d, right). Since ion current is proportional to charge times molar abundance, the observed negative correlation between charging and TIC further indicates a negative correlation between charging and total molar abundance of analytes entering the mass analyzer. One explanation for this observation is the phenomenon of "charge competition": prior work has demonstrated that higher charge availability per analyte (for example from decreasing analyte concentration) resulted in higher charging.[1,2] As such, these results suggest that fluctuations in the total abundance of analytes (against a fixed background of charge availability) cause changes in scan-to-scan peptide charging due to fluctuating levels of "charge competition". Overall, these finding indicate that correlated CSD fluctuations arise from some underlying, time-varying experimental factors.

These findings have potential applications to mass spectrometry identification. Specifically, peptides that have similar CSDs in one scan may exhibit more disparate CSDs in another scan due to differences in charging conditions, similar to what was observed for run-to-run CSD variations (see main text). For example, consider the two co-eluting peptides SNEILTAIIQGMR and ALPFWNEEIVPQIK during the 13 scans shown in Figure S8b. During the low charging scans, the absolute differences in CSD is $\sim$ 7%, whereas during the high charging scans that differences is $\sim$ 14%. Namely, this proof-of-concept example showcases that scan-to-scan charging variations may amplify differences in CSDs, allowing them to be more easily distinguished. Overall, these findings highlight potential avenues for enhancing peptide identification through leveraging scan-to-scan CSD fluctuations.

Supplementary Text 2: Details of CSD extraction scheme.

Here, we provide details about the CSD extraction scheme (see infographic, Figure S9). In this section, the terms "scans" and "spectra" both refer to MS1 mass spectra (at a specific retention time). The term "ion" refers to a peptide combined with a charge state. Adjustable parameters of the extraction scheme are denoted in all caps (default values are provided in "Default parameters" below).

Collapsing ion mobility axis & re-centroiding timsTOF spectra.

Input: Bruker .d folder or .mzML file
Output: centroided spectra (without ion mobility axis)

Here, we describe the steps taken to collapse the ion mobility axis, and re-centroid the resulting peaks for timsTOF MS1 spectra. First, we collapsed the ion mobility axis, retaining the m/z, and intensity axes. Second, we filtered all peaks with intensity lower than CENTROIDING_MIN_PROFILE_PEAK_INTENSITY. Third, we identified peaks that were adjacent in m/z (where two peaks are considered adjacent if they are located consecutively based on the uniform discretization in the "time of flight" scale, that is in the $(m/z)^2$ scale). Third, we grouped peaks based on adjacency (taking the maximum range of peaks that were adjacent to one another). Fourth, for each group of peaks, we assigned one centroided peak, with intensity equal to the sum of intensities and m/z equal to the intensity-weighted average of m/z's.

Processing MaxQuant analysis.

Input: MaxQuant analysis (evidence.txt, summary.txt)
Output: list of ion spectrum pairs, list of peptide spectrum pairs

Here, we describe the steps taken to establish ground truth ions and peptides, and the spectra they are located in, which will be used in the later calibration and extraction stages.

For each identified ion from the MaxQuant analysis, we extracted the start and finish elution times based on those stated in the evidence.txt file. Since each ion may appear multiple times in the evidence.txt file, the start (finish) time was defined as the minimum (maximum) of all occurrences of that ion.

For each identified ion, we paired the ion with all spectra that had a retention time located between the ion's start and finish elution time. For the purposes of quality control, an ion was removed if the start and finish time differed by more than CALIBRATION_MAX_RETENTION_LENGTH.

For each identified peptide, we paired the peptide with all spectra that were previously paired to one of the peptide's ions. In other words, the peptide spectrum pairs are a union of its ion spectrum pairs.

Calibration.

Input: centroided spectra
Output: calibrated spectra

To improve the subsequent extraction stage, we calibrated the m/z axis of the MS1 spectra using the following steps. First, for each ion spectrum pair (see "Processing MaxQuant analysis" above), we computed its m/z offset, given as the difference between the ion's theoretical monoisotopic m/z and its nearest peak in the spectrum. Then, for each spectrum, we calculated a robust average (see below) of all the m/z offsets of paired ions. Lastly, we shifted the m/z of all peaks in the spectrum by that average.

To compute the robust average of m/z offsets, we first computed a rough estimate for the FWHM (full width at half maximum), calculated as 2 median($|m/z$ offsets $-$ median(m/z offsets)$|$). Then, we removed all m/z offsets with magnitude greater than 3 FWHM. Lastly, we took the average of the remaining m/z offsets.

To avoid problematic scans, spectra with an insufficient number of ions were removed from downstream steps, where the minimum number of required ions is determined by CALIBRATION _MINIMUM_REQUIRED_PEPTIDE_COUNT.

Extraction.
Input: calibrated spectra
Output: per-scan CSD readings

Here, we describe the steps taken to extract per-scan CSD readings for each peptide spectrum pair (see "Processing MaxQuant analysis" above). As an overview, peptide CSDs were calculated through normalizing the estimated intensity readings of each charge state. To ensure high-quality extractions, charge state intensity readings were labeled as "confidently present", "confidently absent", or "ambiguous", and CSDs were filtered if they contained any "ambiguous" intensity readings. Below, we describe the specific details of the extraction scheme and the labeling criteria used.

For each peptide spectrum pair, we first computed properties regarding the theoretical and observed isotope distributions. For each charge state from 1^+ to 5^+ (or EXTRACTION_MAX_CHARGE), we computed the first three peaks of the theoretical isotope distribution; we refer these three peaks as #0 (monoisotopic), #1, and #2 peaks, respectively. We also extracted the isotope distribution observed in the spectrum, defined as the collection of the nearest peaks (in m/z) to the theoretical #0, #1, and #2 peaks. From these, we calculated the following properties: the m/z offsets between the theoretical and observed isotope peaks, the cosine similarity between the isotope distributions, and dot product between the isotope distributions (which serves as the charge state's estimated intensity reading). Moreover, we checked for the presence of extraneous peaks that may suggest that the observed isotope distribution overlaps with other peptide spectra. Namely, we extracted the nearest peak to the theoretical #-1 peak (a peak located one neutron below the monoisotopic peak). We also extracted nearest peaks to the theoretical #-1/2, #1/2, and #3/2 peaks (which are located at the midpoints of the #-1, #0, #1, and #2 peaks). Moreover, these extraneous peaks were denoted as non-negligible if their intensities were high (defined as greater than one half of the intensities of adjacent #0, #1, #2 peaks). Lastly, these properties were used to label the given charge state based on the criteria below.

Charge states were labeled as "confidently present" if all three conditions held:

- absolute m/z offset for #0, #1, and #2 peaks < EXTRACTION_MAXIMUM_MZ_OFFSET_FOR_MATCH
- absolute m/z offsets for #-1, #-1/2, #1/2, and #3/2 peaks with non-negligible intensities > EXTRACTION_MAXIMUM_MZ_OFFSET_FOR_EXTRANEOUS_PEAKS
- cosine similarity score > EXTRACTION_MINIMUM_SIMILARITY_SCORE_FOR_MATCH

and consequently assigned an intensity reading derived from the spectrum (see above). Charge states were labeled as "confidently absent" if the following condition held:

- absolute m/z offset for #0 and #1 > EXTRACTION_MINIMUM_MZ_OFFSET_FOR_NO_MATCH

and consequently assigned an intensity reading of zero. Charge states were labeled as "ambiguous" otherwise.

If none of the charge states were labeled as "ambiguous", then the peptide was assigned a CSD reading for the scan, given by the normalized intensity readings. For the results in the main text, each peptide was assigned a single CSD reading through an intensity-weighted average of CSDs across scans.

Default parameters.
The default values for the parameters of the extraction scheme are:

- CENTROIDING_MIN_PROFILE_PEAK_INTENSITY = 100

- CENTROIDING_MIN_CENTROIDED_PEAK_INTENSITY = 500
- CALIBRATION_MAX_ABS_MZ_OFFSET = 0.05
- CALIBRATION_MAX_RETENTION_LENGTH = 5.0
- CALIBRATION_MINIMUM_REQUIRED_PEPTIDE_COUNT = 5
- EXTRACTION_MAX_CHARGE = 5
- EXTRACTION_MINIMUM_SIMILARITY_SCORE_FOR_MATCH = 0.98
- EXTRACTION_MAXIMUM_MZ_OFFSET_FOR_MATCH = $1\times$FWHM
- EXTRACTION_MAXIMUM_MZ_OFFSET_FOR_EXTRANEOUS_PEAKS = $2.5\times$FWHM
- EXTRACTION_MINIMUM_MZ_OFFSET_FOR_NO_MATCH = $5\times$FWHM

where FWHM is determined from the absolute m/z offsets between the theoretical and observed monoisotopic peaks in the "Extraction" stage (e.g., 0.0018 Da for our HeLa trypsin run with 2.5 kV ESI voltage, 160 min gradient length, 400 nL/min flow rate).

References

(1) Wang, G.; Cole, R. B. Mechanistic interpretation of the dependence of charge state distributions on analyte concentrations in electrospray ionization mass spectrometry. *Anal. Chem.* **1995**, *67*, 2892–2900.

(2) Cole, R. B., *Electrospray and MALDI mass spectrometry*, 2nd ed.; Cole, R. B., Ed.; Wiley-Blackwell: Chichester, England, 2010.
